# Blockchain-Based Architecture Design for Personal Health Record: Development and Usability Study

**DOI:** 10.2196/35013

**Published:** 2022-04-13

**Authors:** Thiago Bulhões da Silva Costa, Lucas Shinoda, Ramon Alfredo Moreno, Jose E Krieger, Marco Gutierrez

**Affiliations:** 1 Instituto do Coração Hospital das Clínicas Faculdade de Medicina, Universidade de São Paulo São Paulo Brazil

**Keywords:** electronic health record, personal health record, blockchain, smart contract

## Abstract

**Background:**

The importance of blockchain-based architectures for personal health record (PHR) lies in the fact that they are thought and developed to allow patients to control and at least partly collect their health data. Ideally, these systems should provide the full control of such data to the respective owner. In spite of this importance, most of the works focus more on describing how blockchain models can be used in a PHR scenario rather than whether these models are in fact feasible and robust enough to support a large number of users.

**Objective:**

To achieve a consistent, reproducible, and comparable PHR system, we build a novel ledger-oriented architecture out of a permissioned distributed network, providing patients with a manner to securely collect, store, share, and manage their health data. We also emphasize the importance of suitable ledgers and smart contracts to operate the blockchain network as well as discuss the necessity of standardizing evaluation metrics to compare related (net)works.

**Methods:**

We adopted the Hyperledger Fabric platform to implement our blockchain-based architecture design and the Hyperledger Caliper framework to provide a detailed assessment of our system: first, under workload, ranging from 100 to 2500 simultaneous record submissions, and second, increasing the network size from 3 to 13 peers. In both experiments, we used throughput and average latency as the primary metrics. We also created a health database, a cryptographic unit, and a server to complement the blockchain network.

**Results:**

With a 3-peer network, smart contracts that write on the ledger have throughputs, measured in transactions per second (tps) in an order of magnitude close to 10^2^ tps, while those contracts that only read have rates close to 10^3^ tps. Smart contracts that write also have latencies, measured in seconds, in an order of magnitude close to 10^1^ seconds, while that only read have delays close to 10^0^ seconds. In particular, smart contracts that retrieve, list, and view history have throughputs varying, respectively, from 1100 tps to 1300 tps, 650 tps to 750 tps, and 850 tps to 950 tps, impacting the overall system response if they are equally requested under the same workload. Varying the network size and applying an equal fixed load, in turn, writing throughputs go from 10^2^ tps to 10^1^ tps and latencies go from 10^1^ seconds to 10^2^ seconds, while reading ones maintain similar values.

**Conclusions:**

To the best of our knowledge, we are the first to evaluate, using Hyperledger Caliper, the performance of a PHR blockchain architecture and the first to evaluate each smart contract separately. Nevertheless, blockchain systems achieve performances far below what the traditional distributed databases achieve, indicating that the assessment of blockchain solutions for PHR is a major concern to be addressed before putting them into a real production.

## Introduction

### Background

Two closely related concepts have been drawing the attention of the biomedical and health informatics community: electronic health record (EHR) and health information exchange (HIE). The former, broadly speaking, covers all the repositories of digital data concerning retrospective, concurrent, and prospective information for ongoing support for patient health care [1,2]. Some examples of these digital repositories are electronic medical record [3,4], electronic patient record [5,6], and the personal health record (PHR). In particular, PHR systems are thought and developed to allow health data to be controlled and at least partly collected by the patient [7-9]. The latter, in turn, covers all electronic protocols for transferring data among hospitals, clinics, and other health organizations in order to share standard information regarding patient’s treatment [10]. The Office of the National Coordinator for Health Information Technology defines 3 strategies for HIE: direct, query-based, and consumer-mediated. In particular, consumer-mediated HIE allows patients to retrieve their health information, share it with health care providers and stakeholders they trust, and then make better decisions in partnership [11]. Even though it is a contentious issue yet, patients should ideally have full control of their own health data—authorizing access, sharing, and use—to reach an actual patient-centered HIE [12,13].

Despite having been separately presented, an EHR repository and an HIE protocol can be incorporated into the same system as a matter of fact. In general, they comprise systems to store, retrieve, and share health data and, invariably, lead to interoperability, scalability, reliability, privacy, and security issues regarding those data. Interoperability can reduce or even eliminate handmade administrative tasks, avoid duplicate clinical services, and facilitate access to relevant information, thereby decreasing cost and waste and improving coordinate and unplanned care [14]. Scalability can impact the scale and the transmission of health data, limiting the overall latency and throughput [15]. Reliability can increase confidence in health organizations and contribute to the total testing process, thereby reducing diagnostic errors and supporting malpractice litigation [15,16].

In particular, privacy and security relating to EHRs have been especially important issues because health data are undoubtedly sensitive. Patients must have their personal information guaranteed by civil rights, that is, only used and disclosed under their consent to indeed have privacy. In this sense, health care providers and regulators should be previously authorized before they are able to examine such information. Furthermore, patients must be protected from unauthorized access, modification, and exclusion of their stored data to really be safe. In general, lack of security can result in data theft and leakage [17]. According to the US Department of Health and Human Services Office for Civil Rights, millions of people have had sensitive information stolen and exposed owing to recurrent database attacks on the health care industry [18]. Although traditional cloud-assisted EHR has been a promising paradigm developed to address these issues, cloud environments rely on trusted and centralized third entities, which do not take full responsibility for privacy and security protection and only ensure it as much as possible [19,20]. However, blockchain-based systems, originally created to replace the trusted third party of the financial transactions [21,22], have been spreading to other fields, arousing the interest of the biomedical and health informatics community because they are tightly related to privacy and security concerns over EHR and HIE [19]. Maintaining a distributed, tamper-resistant, and continuously growing ledger, blockchain networks are systems designed to have decentralized storage and management, avoiding the single point of failure and encouraging health care providers and patients to mutually collaborate without the control of a central intermediary. They are also systems created to have a permanent audit trail and a well-defined and consensual set of transaction rules (smart contracts), supplying and certifying health data provenance and establishing formal criteria to handle sensitive information [23-26].

In view thereof, the aforesaid community has already provided an increasing number of blockchain uses: a decentralized record management to handle electronic medical records [27], a PHR smartphone app to empower patients to take control of their own health data [28], an architecture model to provide a PHR in which patients maintain a unified register of their health history even from different organizations [29], a mobile health system to remotely perform cognitive behavioral therapy for insomnia [30], a teledermatology platform to support diagnosis of skin diseases [31], a privacy-preserving location sharing for telecare medical information systems [32], an authentication service to seal biomedical database requests and the respective responses [33], a pharmaceutical supply chain management to prevent counterfeit medicines [34], a framework to share medical images [35], a platform to remotely watch patient vital signs [36], and an EHR to manage and share data from cancer treatment [37], indicating a wide range of promising applications.

### Related Works and Our Contribution

There are several contributions proposing blockchain-based architecture designs to address existing problems with EHR. However, most of them have targeted electronic medical records and electronic patient records, and only few approached PHR [38,39]. Combining traditional database storage, blockchain framework, and smartphone app, Yue et al [28] were among the first to suggest an architecture model to empower the patient’s ability to control and share health data. Despite adopting access control policies in different usage scenarios, the authors did not provide a detailed description of the blockchain infrastructure or perform a system assessment.

Roehrs et al [29] presented a distributed and interoperable model, named the OmniPHR, in which patients can gather their health data to optimally manage their health history and in which health care providers, with the patient’s consent, can access such data, regardless of the institutional source. Although the work pointed out several relevant concepts about the PHR, it only simulated a peer-to-peer network infrastructure using OverSim [40] and did not, in fact, implement a blockchain routine with the timestamped hashing blocks and the smart contracts. To remotely apply cognitive behavioral therapy for insomnia, Ichikawa et al [30] developed a mobile health system based on a Hyperledger Fabric blockchain infrastructure [41] to store the collected data. With a 4-node network, the authors evaluated the tamper resistance under simulated fault by taking 1 node down and subsequently, uploading new data and verifying the information recovery by lifting that node up and, from this, querying the update of the previous data [30]. Even though the work had proposed a PHR system and tested its failure resilience, it did not provide performance indicators—throughput and latency under workload [42-44]—to assess the distributed network infrastructure.

Liang et al [45] developed a mobile app for users to store their personal health data in a cloud database, from wearable or medical devices and manual inputs as well and to share it with health care providers and health insurance companies they trust. Similar to [30], Hyperledger Fabric was the blockchain framework used to implement a permissioned distributed network. Besides Fabric, to improve scalability and integrity, Merkle tree protocol, via Chainpoint [46], was the tree-based data structure used to aggregate hashed records into leaf nodes until reaching a single root—the final hash to be saved in the blockchain. To evaluate performance, the work measured the average time cost during simultaneous recording. In another work, Liang et al [47] elaborated a web application for PHR. The authors built a patient-centered architecture out of a trusted environment, supplied by Intel Software Guard Extension [48] to maintain health data and control access logs regarding these data, and out of a permanent blockchain network supplied by Tierion [49] to record both hashes of that data, certifying integrity and raw copies of that logs, thereby ensuring traceability. To evaluate performance and estimate overload, the work adopted 2 measures: the average time cost to handle a concurrent number of records and the average time cost to handle a large number of access tokens.

Uddin et al [50] proposed an end-to-end eHealthcare architecture for continuous patient monitoring, including a patient-centered component to oversee access control policies, coordinate sensors and devices, and ultimately, decide which data stream should be stored on a blockchain. Inspired by Bitcoin and Ethereum environments [21,51], the authors designed a customized blockchain infrastructure by using Java programming language, with which they implemented a selection of only trusted mining nodes to perform proof of work as consensus protocol. They compared their customized system with Bitcoin’s algorithm performance, analyzing surviving generations value and central processing unit and memory monitoring as metrics [50].

Using an Ethereum-based blockchain network [51], Omar et al [52] developed a privacy-preserving platform in which patients control all health data stored on and retrieved from a blockchain, while having their identity protected by cryptographic functions. Besides that, the authors suggested specific protocols to attain pseudonymity, privacy, integrity, accountability, and security throughout platform transactions. To analyze performance, they evaluated the transaction and execution costs of smart contracts by varying the string length of the data block and employing Ethereum’s crypto-fuel as a metric [52].

Roehrs et al [53] extended the OmniPHR model devised in their prior work to a production scenario, considering a private blockchain network in which only verified and authenticated participants can access and manage it. Notwithstanding Ethereum and Hyperledger Fabric had been pondered as suitable blockchain platforms, the authors preferred to develop their own infrastructure by using open application programming interfaces such as Apache Kafka [54], Apache Zookeeper [55], and others. To evaluate performance over many queries, the work observed how throughput and latency varied from 50 to 500, from 1000 to 10,000, and from 13,000 to 40,000 concurrent requests.

Through an Ethereum-based blockchain architecture, Lee et al [56] proposed an international cross-area platform to arrange data from different health care services and manage authorizations for HIE among patients, health care providers, and stakeholders. By considering a test scenario in which a person had traveled from her/his home country to a foreign one and suddenly needed medical attention, the patient, registered on the platform, successfully granted a physician authorization to access her/his PHR. The physician, in turn, also registered on the platform, searched the requested PHR, and according to it and the current patient condition, provided a diagnosis and ordered treatment and medication [56].

Alongside the preceding papers, our work builds a blockchain-based architecture out of a permissioned distributed network in order to supply a PHR system for patients to securely collect, store, share, and manage their health data. Despite the similarities, it brings a novel ledger-oriented architecture model using Hyperledger Fabric, emphasizing the importance of suitable ledgers and smart contracts to operate the overall blockchain. In addition, it provides a detailed assessment of a 3-peer network—applying throughput and latency—under workload, ranging from 100 to 2500 simultaneous record submissions, and analyses, in this case for a fixed load, the impact of increasing the network, ranging from 3 to 13 peers. At the end, our work discusses the necessity of standardizing evaluation metrics to facilitate the comparison between related works.

## Methods

### Blockchain and Smart Contracts

Blockchain is a distributed, tamper-resistant, and continuously growing ledger for recording desirable assets and transactions in cryptographically chained blocks. It results from a protocol to add data blocks, using public-key cryptography and hash functions, and from a protocol to validate them, using a consensus algorithm on a peer-to-peer network [21]. In this sense, each new block contains the timestamp, the hash of the previous block, and the list of the retrospective and current digitally signed assets and transactions. Each new one is also verified by the majority of the peers in order to provide a reliable full history of the register. Once the assets and transactions are validated by consensus, the new block is recorded in the chain and becomes immutable. Subsequently, the updated ledger is shared by all peers and, thenceforth, can be attested without the need of a central authority [57,58].

Blockchain networks can be arranged either into a permissionless or a permissioned mechanism for selecting participants, to ensure the honest majority assumption, that is, the conjecture that the majority of the peers will be honest and run the consensus protocol correctly [59]. On the one hand, a permissionless blockchain network—a domain of the cryptocurrencies and financial markets [60]—does not have administrators managing membership or banning illegitimate peers; it is literally open to anyone who wants to be part of it [58,61]. In these circumstances, the network maintains incentive alignments as long as participants self-select but must expend computational resources, as in the proof of work, or even money, as in the proof of stake, to run the consensus protocol [59]. On the other hand, a permissioned blockchain network—a domain of the business and institutional practices [60]—has external administrators managing membership and defining which peers have read and write permission on the blockchain [58,61]. Although choosing the participants is outside the scope of the consensus protocol, the network establishes a consortium whereby members obey publicly documented policies to achieve group decision-making [59].

Smart contracts, in turn, are prespecified rules that allow a blockchain to be conducted in a consensual manner by all network participants. In practice, these rules represent transactions, which automatically operate digital assets and can be constructively used to state a bylaw among parties with common goals, attaining a decentralized autonomous organization [51]. Encoding state transition functions, smart contracts are logically and effectively implemented as executable programs in both domain-specific and general-purpose languages and owe their security to the accomplishment of the consensus protocol [41]. Despite opening a way to make digital codes into laws or official statements, blockchain and smart contracts are emerging technologies still. Therefore, they neither are legally binding documents nor have a jurisprudential agreement to be interpreted [61].

As already suggested in the introduction, Ethereum and Hyperledger Fabric have been the main open-source platforms used to develop blockchain frameworks into EHR and HIE [23-26,38,39]. Providing a built-in, Turing-complete, and domain-specific language (Solidity) to write smart contracts and distributed applications, Ethereum is an alternative to the first-generation scripting systems without full programming capabilities [51]. In the beginning, it was launched to create permissionless networks [62], implementing a consensus protocol (Ethash) based on the proof of work, in which a hash puzzle needs to be solved by a prover and validated by a set of verifiers [22]. To mediate this computation and avoid network abuse, Ethereum has an internal cryptocurrency (Ether) to charge transaction fees and reward nodes competing to append new blocks to the chain [63]. By the advent of the permissioned networks, Ethereum was also adapted to support general purpose languages such as Go and C++ [23] and run a consensus protocol based on the proof of authority, in which only a set of known verifiers can be selected to validate a new block [22].

Hosted by the Linux Foundation, Hyperledger Fabric, in turn, is a decentralized operating system to create permissioned networks. It allows smart contracts (chaincodes) and distributed applications to be written in Go, Java, and Node. Using an ordering service implementation based on a crash-tolerance consensus [22], it has an endorsement policy in which the smart contracts themselves, via chaincode lifecycle and private communication mechanisms (channels), specify a set of nodes to endorse transactions. In this sense, the nodes in Hyperledger Fabric have different functions: the client nodes to propose, orchestrate, and broadcast transactions, the peer nodes to execute and validate transactions as well as to maintain the ledger and the smart contracts, and the ordering service nodes to mediate state updates and dependencies during transaction execution. To control the identity of these nodes, Hyperledger Fabric has a membership service provider to handle certificate authorities and public key infrastructure and, from them, issue credentials for authentication and authorization [41,62].

As already mentioned, we opt for the latter platform to implement our permissioned network. Most of the existing platforms, including Ethereum, implement a traditional active replication for the consensus protocol, which first orders and broadcasts transactions to all peers and second waits for each peer to perform such transactions sequentially (order-execute paradigm), limiting performance and requiring an additional mechanism to prevent denial-of-service attacks from untrusted codes [41]. Executing transactions only on a subset of peers, Hyperledger Fabric implements an execute-order-validate paradigm, which first performs and verifies the transactions, then orders through a consensus protocol, and finally validates such transactions by the application-specific trust assumptions [41]. Although there are scalability issues, Hyperledger Fabric has indeed exhibited better throughput and latency values than Ethereum and other blockchain platforms [42,43,62]. In addition to these characteristics, it provides an entire set of privacy-preserving mechanisms to create and submit private transactions [41,62]—a decisive quality that influenced our decision.

### Blockchain-Based Architecture Design for PHR

Using Hyperledger Fabric release 2.2, our blockchain network is structured with N peer nodes (P1, P2, …, PN), with N greater than or equal to 3, and an ordering service node. The peer nodes are the basic elements of the network because they store ledgers (L) and smart contracts (S) [64]. Ideally, each peer infrastructure must be under the responsibility of a different corporation. In this sense, they can represent N interested parties—the government, health organizations, civil society institutions, hospitals, among others—acting for the maintenance and evolution of a PHR. Thus, the peer nodes provide network services such as the writing and reading of the ledgers for administrators and users relating to these parties. In theory, there is no upper bound for N other than that imposed by the hardware and software running the consensus protocol. In this sense, we first investigate a 3-peer network because it is the smallest one in which the majority assumption is reasonable and, second, analyze the impact of increasing N.

The peers are associated with their respective client nodes (CL1, CL2, …, CLN)—the elements outside the network that allow an application to be connected to the blockchain, that is, an external application accesses ledgers and smart contracts via client-peer connection. By means of a software development kit [65], Hyperledger Fabric supplies an application programming interface with instructions to perform the aforementioned connection in order to submit transactions as well as to receive responses after these transactions are finished or interrupted earlier due to the lack of consensus. In addition, Hyperledger Fabric conceives of a channel (C) as a primary communication pathway by which peers and clients can establish a consortium with well-defined policies, thus providing a mechanism for isolating assets and transactions from the rest of the network. In this context, each smart contract and the respective ledger can be separately invoked on a specific channel only by users previously registered in the consortium, thereby ensuring interoperability and privacy [64].

The peers get assigned to the consortium—the government, health organizations, civil society institutions, and hospitals in our example—by their respective certificate authorities (CA1, CA2, …, CAN), the elements that generate public and private key infrastructure to issue identities via digital certificates [66]. Hyperledger Fabric has adopted the X.509 standard [67] as its primary certificate system. Whenever one of the consortium members establishes a client-peer connection to access the blockchain resources, these certificate authorities attest to the channel the digital identity of the applicant and her/his rights to use the required smart contract. As already mentioned, the Fabric component mapping identities with their own rights is the membership service provider, which inspects who participates in the network and their channels, identifying roles and limits of all administrators and users [64].

**Figure 1:**
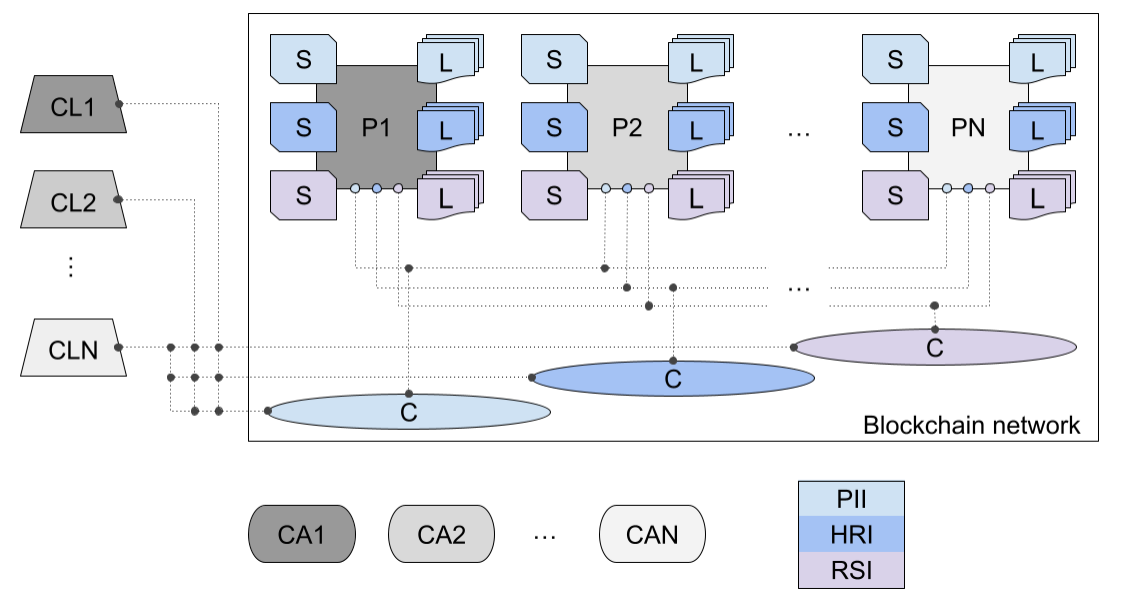
Design of our blockchain network, considering N endorsement peers and their respective clients and certificate authorities. Each channel is associated with a specific set of ledgers and smart contracts, respectively named as personally identifiable information, health record information, and record sharing information. Ideally, each triple peer-client-certificate authority must be under the responsibility of a different organization or institution. HRI: health record information; PII: personally identifiable information; RSI: record sharing information; P: peer; S: smart contract; L: ledger; CL: client; CA: certificate authority; C: channel.

Lastly, the ordering service node mediates the interaction between peers during a transaction submission and ensures a consistent ledger after performing the consensus protocol. In Hyperledger Fabric, the endorsement policy occurs as a result of a 3-phase process: (1) proposal, (2) ordering and packing, and (3) validation and commit. Roughly speaking, in the first phase, a client node submits a transaction proposal, which is distributed to the endorsement peers and is independently executed by them, returning a set of endorsed responses—inconsistent responses can be already detected and discarded, finishing the workflow early. In the second phase, the ordering service node collects these responses and packages them into blocks, preparing for the next step. In the third phase, the ordering service node finally distributes the blocks to the peers, which in turn validate them to verify the endorsement phase and, only after that, commit to the ledger—failed transactions terminate the workflow without writing on the blockchain [64]. Figure 1 summarizes our architecture design, just omitting the ordering service node for a better visualization. The N peers in our network are configured to participate in the endorsement phase.

**Figure 2:**
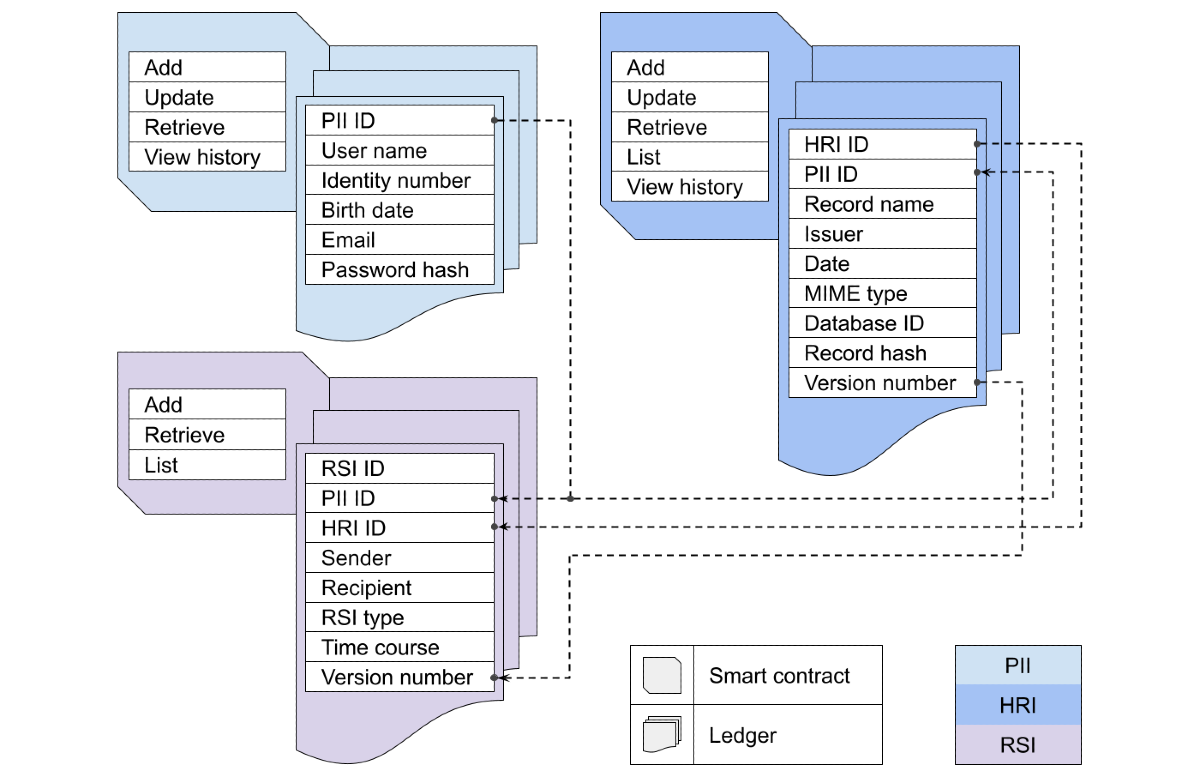
Design of the ledgers and their respective smart contracts. They fall into 3 classes: personally identifiable information, health record information, and record sharing information. HRI: health record information; MIME: Multipurpose Internet Mail Extensions; PII: personally identifiable information; RSI: record sharing information.

Turning the analysis to the ledgers and smart contracts, our approach considers 3 classes: (1) for personally identifiable information (PII), (2) for health record information (HRI), and (3) for record sharing information (RSI) (Figure 2). By opting for 2 or more ledgers (3 in our case), blockchains also evolve in an intricate and unpredictable way, which makes any attempt to tamper with health records even more difficult and unlikely as long as the system is in use. Besides the tamper resistance, such configuration permits the blockchain network to be structured in an oriented-ledger architecture design, making data organization aligned with the resource consumption.

PII is designed to store basic form data filled by the user at the moment of registration in the system. There are smart contracts to add, update, retrieve, and view history, respectively, to write a new record, rectify a registration error, perform a system login, and recover an updating log. To add a PII, the user needs to register with a password—converted into a hash value for security—and thus, receive a unique identifier (PII ID). Once registered, the PII ID is only recovered from a login, that is, identity number or email and the correct password hash. All other smart contracts, including those from HRI and RSI, are only able to write and read the ledger by means of a PII ID as the prefix of a composite key. In such a way, each user just accesses her/his data. HRI, in turn, is designed to store metadata from a health document, together with a hash value and a database ID, for reasons to be explained later in the text. Similar to the PII, there are smart contracts to add, update, retrieve—in this case, to recover a single record—and view history, and one further to list all records for a user. Finally, RSI is designed to store HIE logs in order to track every time a copy of a health document leaves the repository, either for downloading or sharing. There are smart contracts to add, retrieve, and list. To keep HIE logs unchanged, we opt for not creating a smart contract to update them; hence, neither one to view history.

Notwithstanding the necessity of smart contracts to list HRI and RSI, for the sake of security, PHR systems do not need one to list PII. One such smart contract would allow an administrator to list users and associate them with their respective HRI and RSI. To prevent such a situation and actually grant to a user the exclusive right of her/his health data ownership, the PII ID is only retrieved with the correct password hash. Because PII ID is a required index prefix to use HRI and RSI smart contracts, the absence of a PII listing function represents an additional security element directly configured in the operation rules of the system. Note that these settings are not just programming practices. Because smart contracts state the logic of the blockchain network, a set of security practices at the present time can evolve to rule status in the near future. Indeed, using smart contracts is a great opportunity to create a bylaw or business logic for PHR, defining which is and is not permitted regarding the access to patient information.

Although there are several smart contracts, they consist of 2 basic network operations: writing and reading. The former is used to invoke either the creation of a new state on the ledger or the modification of an existing one—without deleting past states, evidently. Smart contracts to add and update fall into this type. To perform writing, a client node needs to start an endorsement policy and reach consensus—a process that involves all peers. The latter operation, in turn, is used to query the current state and history of a ledger. Smart contracts to retrieve, list, and view history fall into this another type. To perform reading, a client node just connects to its associated peer and thus queries the stored ledger, independently of the other peers. Similar to the client-peer connection resources, by means of another software development kit [68], Hyperledger Fabric supplies an application programming interface with instructions for the development of smart contracts and business logic. As already mentioned, Fabric provides support for Go, Java, and Node, but we adopt the latter as our primary programming language to build our architecture design.

### Health Database, Cryptographic Unit, and Server

Although blockchain technology provides security tools against record tampering, it is still not suitable for storing a large volume of data, despite the efforts made to meet this requirement [69]. Nowadays, only metadata such as PII, HRI, and RSI can be recorded and maintained in a blockchain network. Therefore, our system also includes a NoSQL database to permit the scaling of all sorts of health data (text, signals, and images) in clusters of machines. To implement our NoSQL health database, we adopted MongoDB, a document-oriented database, which indeed supports methods to distribute and replicate data across multiple machines and provides lower execution times than a relational one, making the scaling out easier for applications demanding both a large volume of data and a large number of queries [70]. In summary, while metadata (PII, HRI, and RSI) are stored on the blockchain network, data, that is, digital health documents, are stored on a distributed health database as soon as the network achieves consensus. In these circumstances, the health documents are hashed and their hash values are included as metadata in HRI to shield them from breaches. Note that the blockchain network represents an audit system [71] and the health documents can be anonymized in the health database, apart from a database ID in the sole possession of the user.

As a further safeguard, the data and metadata are encrypted. When a user registers in our system, she/he automatically receives a key to encrypt information entering the system as well as to decrypt that leaving out by means of a cryptographic unit. Each user obtains her/his own key and is only capable of decrypting her/his own data evidently. Because our health database is configured to store documents smaller than or equal to 100 MB, we opt for using the advanced encryption standard (AES), a symmetric key block encryption algorithm recommended by the National Institute of Standards and Technology. The AES handles block sizes of at least 128 bits and key sizes of 128, 192, and 256 bits. The AES also accepts 5 modes of operation, that is, electronic codebook, cipher block chaining (CBC), cipher feedback, output feedback, and counter, for preventing identical ciphertexts to be generated from blocks containing the same data, a breach that facilitates a malicious opponent to accumulate enough plaintext-ciphertext pairs and thus find the key by exhaustion in a feasible time. In particular, CBC requires an initialization vector, which takes an exclusive-OR operation with the first plaintext block and, if randomly generated, provides different ciphertexts from the same data [72,73]. We adopt CBC as our mode of operation and 256 bits as our key and initialization vector sizes, resulting in the AES-256-CBC algorithm. The key and initialization vector of each user are allocated in a private wallet/folder, alongside her/his digital certificate.

**Figure 3:**
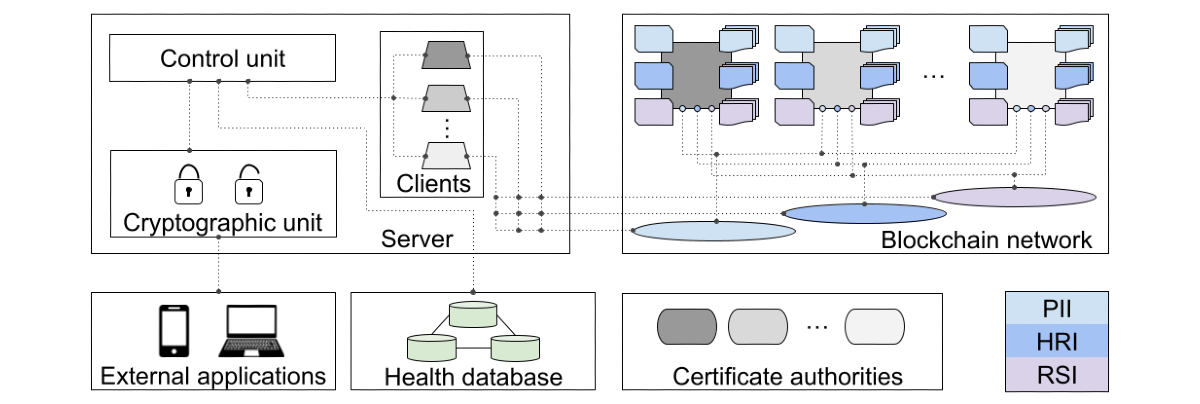
Sketch of the overall system, exhibiting the interconnections between server, health database, and blockchain network, in order to provide personal health record resources for external applications. HRI: health record information; PII: personally identifiable information; RSI: record sharing information.

**Figure 4:**
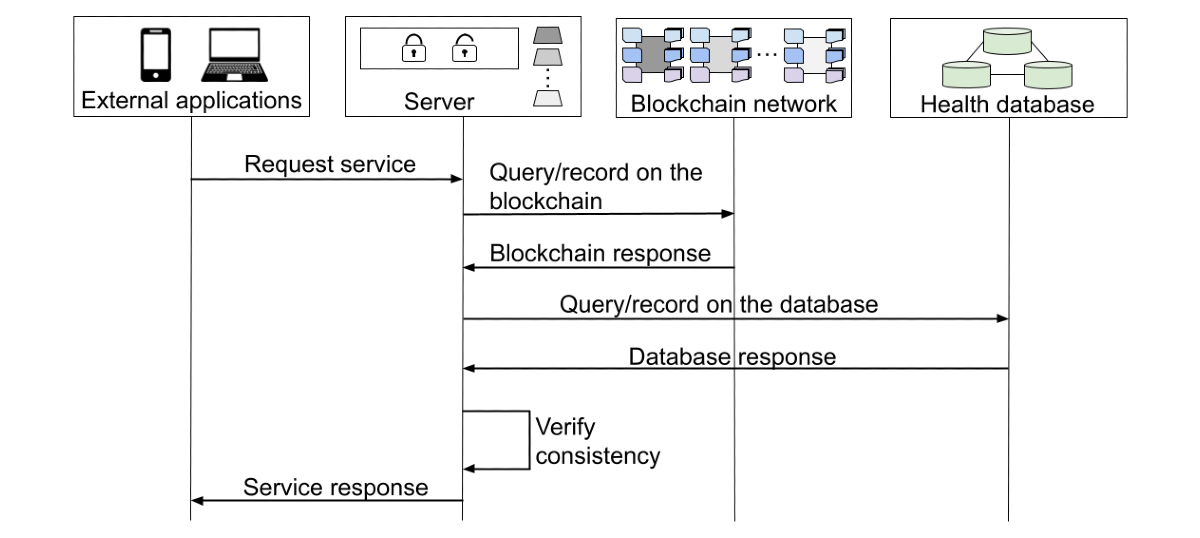
Flow of information during the query or record request of a health document. The server only returns a successful response if data and metadata are consistent. The flow can be interrupted earlier owing to lack of consensus.

As a final module, we build a server infrastructure out of a Node framework to host the blockchain clients and, thereby, provide blockchain resources for external applications. Through a control unit, and performing specific calls for each smart contract as well as for each database operation, this server supports the registration and access of users, the inclusion, updating and retrieval of health documents, and the creation of links to download and share these documents—only with the consent and supervision of the respective user, evidently. Roughly speaking, this server executes 3 basic steps: (1) it receives requests from external applications, (2) according to each request, it accesses the corresponding network and database resources, and (3) it returns consistent responses to those applications. Because the server works as an intermediate system between blockchain network, health database, and external applications, it conveniently accommodates the cryptographic unit. In this way, sensitive information is encrypted as soon as it enters the system and only decrypted when leaving out. Figure 3 highlights all these interconnected modules and Figure 4 exemplifies the flow of information during the query or record request of a health document.

### Evaluation Benchmark

To evaluate our blockchain-based architecture design, we use Hyperledger Caliper—a benchmark tool released by the Hyperledger community for measuring the performance of blockchain systems and producing reports containing metrics commonly accepted, such as throughput and latency. Caliper supports Ethereum and Hyperledger Fabric, allowing computer scientists and engineers to compare EHR proposals developed from the 2 main platforms at present. It is capable of generating a workload for a system under test (SUT) and continuously monitoring responses from this SUT [44,74].

To run an experiment, Caliper requires a benchmark file, a network file, and workload modules. The first one presents custom configurations to run the benchmark, such as the number of workers to perform a workload, the round settings, the number of submissions, the round length in seconds, the rate at which transactions are sent to the blockchain, among others. The second one presents the layout of the SUT—basically, the addresses and identities of the nodes and the channels and smart contracts to be used during the test. Lastly, workload modules are Node functions exported to simulate client nodes sending requests to the SUT, that is, in each round, a different workload module can be used to generate and submit transactions to the SUT, according to the configurations in the benchmark and network files. Therefore, Caliper can emulate many clients injecting workloads in a blockchain network [44,74].

As already mentioned, 2 basic metrics to assess blockchain performance are throughput and latency. The former, usually given in transactions per second (tps), represents the total number of valid transactions reached in a period of time [42]. In this sense, invalid transactions are subtracted from the total to yield the valid ones. Because transactions fall into reading and writing operations, throughput also falls into these types. On the one hand, reading throughput may be informative, but it only measures operations taken on a single client-peer connection, independently of the other peers, and therefore, is not a primary measure. On the other hand, writing throughput considers operations invoking the consensus protocol and thus committing transactions at all endorsement peers, making it much more informative than the preceding rate [75].

The latter, in turn, usually given in seconds, represents the time taken for a transaction to conclude and return a response [42]. Similar to the throughput, latency also falls into reading and writing types: the first one measures delays from a single client-peer connection, while the second one from all endorsement peers. In particular, writing latency includes the propagation and settling times due to the consensus protocol, considering delays measured over the entire network. Although this metric is generally calculated per transaction, the average latency is more suitable to assess blockchain performance [75].

## Results

With a 3-peer network, our first benchmark is set to run a workload, from 100 to 2500 simultaneous submissions of health metadata, with steps of 100, on each smart contract of the PII, HRI, and RSI templates. We limit our test to 2500 loads because Hyperledger Fabric is standardly configured to perform a maximum of 2500 concurrent requests. Writing scenarios are configured to use 5 workers submitting at the same time 10,000 transactions, each one totalizing 50,000. Reading scenarios are configured to use the same 5 workers in parallel but to randomly request records during 600 seconds of continuous operation. The rate controller is kept in a fixed-load mode, starting at 50 tps and 500 tps, for writing and reading transactions, respectively, and growing to reach maximum rates. Because PII, HRI, and RSI are designed to store ciphertexts only, in our test, all simulated submissions of health metadata are randomly generated as strings of fixed length for each smart contract field. An empty blockchain network is raised in each load test to guarantee an equal condition. Our test environment consists of a machine having an Intel Xeon E-2246G processor (12 MB cache, 3.60 GHz, 6 cores, 12 threads), an NVIDIA Quadro P1000 graphic adapter, and a random access memory of 16 GB, running Ubuntu 18.04.5 LTS 64 bits operating system.

**Figure 5:**
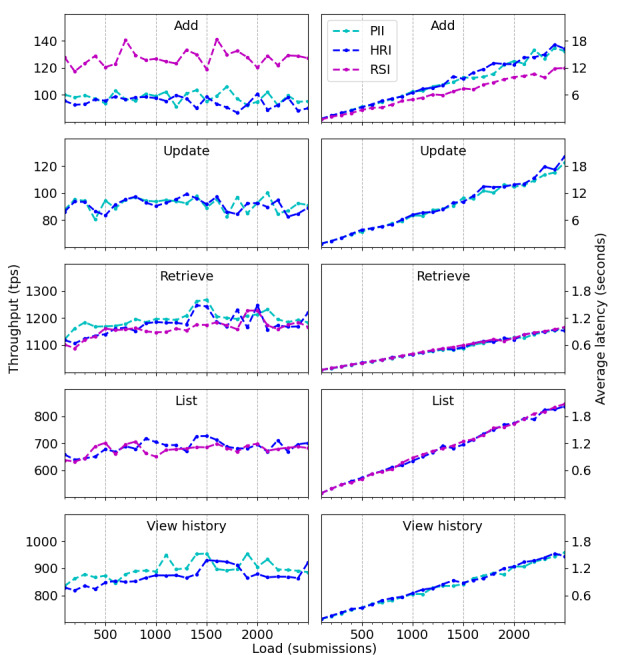
Throughput (measured in transactions per second) and average latency (measured in seconds) of all smart contracts under workload, ranging from 100 to 2500 concurrent submissions of health metadata, with steps of 100. HRI: health record information; PII: personally identifiable information; RSI: record sharing information; tps: transactions per second.

Figure 5 exhibits the throughputs and average latencies in relation to PII, HRI, and RSI smart contracts under workload. We do not report transaction errors because not one occurred. Disregarding the small variations inherent in each workload trial, and albeit with different baselines, the throughputs of all smart contracts remain fairly constant over the interval, a consistent behavior given that the system responses appear to be invariant to load. Smart contracts to add and update a record have rates with an order of magnitude close to 10^2^ tps, while those to retrieve, list, and view history have rates close to 10^3^ tps. As already suggested, this difference arises mainly because writing transactions trigger the consensus protocol, mobilize the network as a whole, and then need more time to process all submissions, whereas reading ones only involve a single client-peer connection. Although with different upward slopes, the average latencies of all smart contracts present a linear growth as workload range varies, a reasonable behavior inasmuch as an increase in submissions demands a proportional increase in processing. In this case, smart contracts to add and update a record have delays with an order of magnitude close to 10^1^ seconds, while those to retrieve, list, and view history have delays close to 10^0^ seconds. In analogy with the throughput, there is an obvious difference between writing and reading transactions, for the same reason as before.

**Figure 6:**
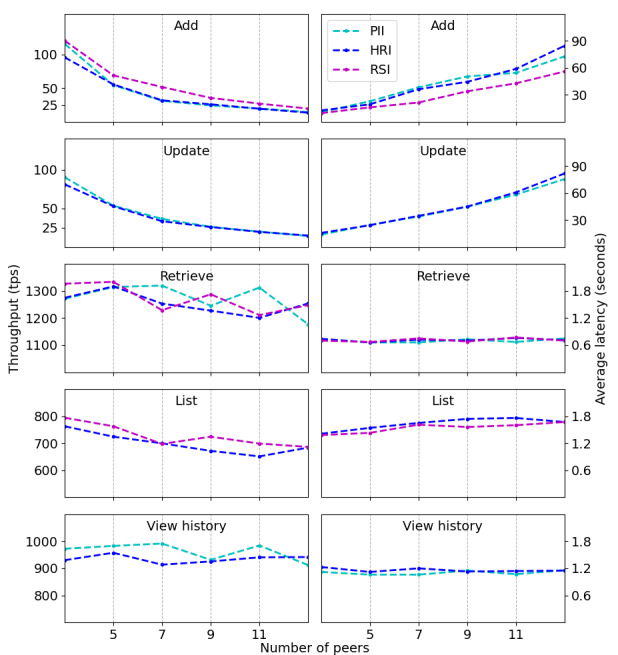
Throughput (measured in transactions per second) and average latency (measured in seconds) of all smart contracts, by considering a network increase from 3 to 13 endorsement peers, with steps of 2. HRI: health record information; PII: personally identifiable information; RSI: record sharing information; tps: transactions per second.

Even though throughputs of reading transactions present a similar order of magnitude, they have significant differences between them. Smart contracts to retrieve, list, and view history have throughputs varying, respectively, from 1100 tps to 1300 tps, from 650 tps to 750 tps, and from 850 tps to 950 tps. Their latencies, in turn, grow at slightly different linear rates, albeit alike. These 2 pieces of evidence suggest that reading transactions can impact the overall system response if they are equally requested. An external application under a real situation has to consider the smallest of these values as the upper limit to avoid overload. With a fixed load at 2000 submissions, our second benchmark is set to increase the network size from 3 to 13 peers, with steps of 2, and perform, for each case, the writing and reading scenarios of the previous experimental protocol. We limit the largest network to 13 peers because by considering our test environment, Hyperledger Fabric has a very poor performance beyond this value, resulting in many transaction failures. Figure 6 displays the throughputs and average latencies when the size of the network increases. For reading smart contracts, they remain fairly constant over the interval, sustaining orders of magnitude close to 10^3^ tps and 10^0^ seconds, respectively, a consistent behavior given that such operations rely on a single client-peer connection. Writing smart contracts, in turn, start with throughputs close to 10^2^ tps but end with rates close to 10^1^ tps, exhibiting an exponential decay. They also start with latencies of 10^1^ seconds but end with delays of 10^2^ seconds, presenting a linear growth. Both pieces of evidence corroborate the well-known scalability issue of Hyperledger Fabric when the number of endorsement peers increases.

As a final comment when observing throughputs and average latencies in Figures 5 and 6, despite the obvious differences regarding each smart contract operation (to add, update, retrieve, list, and view history), the ongoing metrics of the 3 proposed templates (PII, HRI, RSI) do not reveal large deviations within a single operation, indicating a similar performance even with slightly different sizes of health metadata.

## Discussion

The results of this study are comparable to those reported previously in the literature [42-44], indicating that blockchain systems achieve performances far below what the traditional distributed databases achieve [76,77]. Traditional databases make use of concurrency control, for example, 2-phase locking to ensure atomicity, consistency, isolation, and durability. By and large, they exhibit better performance because they consider simple failure models such as crash failure. Oppositely, blockchain systems consider Byzantine failure and, in the worst scenario, a hostile environment in which nodes can join and leave the network, which undeniably makes the overhead of concurrency control much more difficult to handle [42]. However, despite being widely recommended by the blockchain community [42-44], throughput and latency have not been commonly adopted metrics for evaluating PHR. Yue et al [28] did not even perform a system assessment; Roehrs et al [29] only simulated a peer-to-peer network and then, provided an inferred latency; Ichikawa et al [30] assessed the tamper resistance in a fault simulation context; Liang et al [45] and Liang et al [47] measured an average time cost to handle simultaneous records; Uddin et al [50] employed surviving generations value as well as central processing unit and memory monitoring; Omar et al [52] opted for Ethereum’s crypto-fuel; and Lee et al [56] proposed a test scenario in which a person and a doctor actually used the system [56]. Only Roehrs et al [53] observed how throughput and latency varied, under workload, from 50 to 500, from 1000 to 10,000, and from 13,000 to 40,000 concurrent requests as light, medium, and heavy scenarios, respectively. The authors achieved, in the heavy one, impressive values: 2298 tps and 0.404 seconds on average [53]. However, the authors arranged health data on single data blocks with writing and reading capabilities as a unified view of patients, thus not performing a bylaw or business logic for PHR and only assessed reading transactions considering these blocks. Furthermore, they did not develop their network from an open-source platform, hindering system reproducibility.

In practice, most of the works focus more on describing how blockchain models can be used in a PHR scenario than whether these models are in fact feasible to support a large number of users. Because the health industry can easily cover tens or even hundreds of millions of patients in a single country, we think the assessment of blockchain solutions for PHR is a major concern to be addressed before putting them into a real production. In view thereof, there is a latent necessity of standardizing evaluation metrics to facilitate the comparison between related works. We think that throughput and average latency are suitable metrics for this purpose as well as Hyperledger Caliper and BLOCKBENCH [42] adequate frameworks to perform this evaluation.

Toward a consistent, reproducible, and comparable PHR evaluation, and by regarding throughput and latency, we are the first to evaluate with Hyperledger Caliper the performance of a PHR blockchain architecture. Because Caliper is the official benchmark to access blockchain networks built out of Fabric, we believe that our results bring important insights to the limits and advantages of using Fabric to design PHR repositories. Moreover, Caliper can be adapted to access Ethereum-based systems, facilitating the comparison between architectures created with the 2 main open-source platforms at the present time. To the best of our knowledge, we are also the first to evaluate each smart contract separately. Previous works considered smart contracts as falling only into writing and reading transactions and have just identified dissimilarities between these 2 types. However, we reveal that, especially in relation to reading ones, throughput and latency can have significant differences, impacting the overall system response if these transactions are equally requested under the same workload.

Specifically in relation to our proposal, as a first implementation, the blockchain network, the health database, and the server are allocated through virtual machines on a single physical device, only simulating a decentralized system, which represents a limitation of our work. Furthermore, because we are primarily interested in the blockchain architecture, the health database and the server are incorporated in the model but they are not actually tested considering an external application under a real situation, which represents an additional limitation. We leave these improvements for future work because we believe that our current results already provide important advice to the biomedical and health informatics community.

In conclusion, the importance of blockchain-based architectures for PHR lies in the fact that they are thought and developed to allow a patient to control and at least partly collect health data, as well as to share health information on her/his own. Ideally, these systems should provide the full control of such data for the respective owner [78]; that is, each patient must authorize health care providers and stakeholders (s)he trusts before they can access her/his personal health data. Exactly because blockchain systems are tightly related to privacy and security concerns, several works are proposing blockchain-based solutions to the health care industry. In line with these efforts, we build a novel ledger-oriented architecture out of a permissioned distributed network in order to support a PHR system for patients to securely collect, store, share, and manage their health data. We emphasize the importance of suitable ledgers and smart contracts to operate the overall blockchain network and provide a detailed assessment of this network under workload, ranging from 100 to 2500 concurrent submissions, and increasing the network size from 3 to 13 peers. To the best of our knowledge, we are the first to evaluate with Hyperledger Caliper the performance of a PHR blockchain architecture and the first to evaluate each smart contract separately. However, our system elements are allocated through virtual machines on a single physical device, only simulating a decentralized system. Besides this limitation, our health database and server are incorporated in the model but they are not actually tested considering an external application under a real situation. We intend to perform these enhancements in future works.

## Abbreviations

AES: advanced encryption standard
CBC: cipher block chaining
EHR: electronic health record
HIE: health information exchange
HRI: health record information
PHR: personal health record
PII: personally identifiable information
RSI: record sharing information
SUT: system under test
tps: transactions per second
